# Metastatic Bone Disease Is Associated With Longitudinal Muscle Loss and Reduced Survival in Kidney Cancer

**DOI:** 10.1002/jcsm.70400

**Published:** 2026-10-01

**Authors:** Jon‐Luc Poirier, Samuel T. Jines, Libbie M. Silverman, Anuradha Valiya Kambrath, L. Daniel Wurtz, Teresa A. Zimmers, Christopher D. Collier

**Affiliations:** ^1^ Department of Orthopaedic Surgery Indiana University School of Medicine Indianapolis Indiana USA; ^2^ Department of Surgery Indiana University School of Medicine Indianapolis Indiana USA; ^3^ Simon Comprehensive Cancer Center Indiana University School of Medicine Indianapolis Indiana USA; ^4^ Indiana Center for Musculoskeletal Health Indiana University School of Medicine Indianapolis Indiana USA; ^5^ Department of Cell, Developmental & Cancer Biology Oregon Health & Science University Portland Oregon USA; ^6^ Knight Cancer Institute Oregon Health & Science University Portland Oregon USA

**Keywords:** bone loss, cancer cachexia, metastatic bone disease, muscle loss, renal cell carcinoma, survival

## Abstract

**Background:**

Cachexia is a multifactorial muscle wasting syndrome and is responsible for 20%–40% of cancer‐related deaths, yet no FDA‐approved therapies exist. Preclinical studies suggest that metastatic bone disease may contribute to muscle wasting, but this relationship has not been evaluated in clinical populations. This study evaluated whether metastatic bone disease at diagnosis is associated with longitudinal skeletal muscle loss and whether metastatic bone disease and longitudinal skeletal muscle loss are associated with overall survival in patients with kidney cancer.

**Methods:**

A total of 293 patients (66.9% male, 33.1% female, median age 60 years) were retrospectively identified with either localised kidney cancer, extraosseous metastatic disease, or osseous metastatic disease at diagnosis. Clinical and demographic variables and treatments were recorded. Body composition was quantified over time for a subset by body mass index (BMI) (*N* = 221) and CT analysis at the L3 vertebral level (*N* = 99). Longitudinal changes were analysed using linear regression, and overall survival was assessed with Kaplan–Meier and multivariable Cox proportional hazards models.

**Results:**

Patients with osseous metastatic disease had a reduction in BMI over time compared with those with localised or extraosseous metastatic disease (median of −3.6% per year, compared with +0.7% and −2.6% respectively, *p* < 0.01). Skeletal muscle loss was greatest for those with osseous metastasis (−7.5% per year, compared with −0.8% and −1.2% respectively, *p* < 0.01). Multivariable linear regression demonstrated osseous metastasis at diagnosis was strongly associated with muscle loss (*β* coefficient of −20.9 [95% confidence interval (CI): −35.56 to −6.27]). In multivariable Cox regression analysis, osseous metastasis at diagnosis was associated with an increased risk of death (HR 15.8; 95% CI: 2.00 to 124.95; *p* < 0.01), as was longitudinal skeletal muscle loss (HR 1.02 for each 1% loss per year; 95% CI: 1.01 to 1.04; *p* < 0.01).

**Conclusions:**

This study provides the first clinical evidence that metastatic bone disease is strongly associated with skeletal muscle loss and reduced survival in kidney cancer patients. These findings extend prior preclinical observations into the clinical setting and support further investigation into the possibility that tumour–bone–muscle interactions drive systemic muscle wasting. Importantly, the observation that severe muscle loss is strongly associated with mortality in kidney cancer highlights the need to consider cachexia as a modifiable determinant of outcomes, not just a byproduct of advanced disease.

## 1 Introduction

Cachexia is a wasting syndrome characterised by systemic loss of muscle and bone mass that affects up to 80% of patients with advanced cancer [1]. It impairs skeletal, diaphragmatic and cardiac muscle function [2]; reduces tolerance to systemic therapy; and accounts for 20%–40% of cancer‐related deaths [3]. Despite this clinical impact, relatively little is known about its pathogenesis, and there are no FDA‐approved agents for its treatment despite new therapies such as the GDF‐15 inhibitor ponsegromab showing promise [1, 4]. As cancer survival improves, the burden of cachexia is increasing, underscoring the urgent need to identify disease‐specific drivers and develop effective therapies [3].

Metastatic bone disease is a frequent complication of cancer, present in 30% of patients with metastatic disease at diagnosis and up to 70% at autopsy [5]. Although management typically focuses on palliation of pain and fracture risk, growing evidence suggests that bone metastases have systemic consequences, including cachexia. Tumour–bone interactions invigorate metastatic growth [6] and release bone‐derived factors that promote muscle wasting [7]. These findings support the hypothesis that metastatic bone disease may directly contribute to the development of cachexia, but this relationship has not been rigorously evaluated in clinical populations.

Kidney cancer provides an ideal setting in which to address this knowledge gap. Both cachexia and bone metastasis are common in kidney cancer, with approximately 40% of patients developing cachexia [1] and 30%–40% experiencing skeletal involvement during the disease course [5]. Clear cell renal cell carcinoma (RCC) accounts for nearly 90% of kidney cancer cases [8], and prior studies in RCC have shown that low skeletal muscle mass is associated with worse survival [9]. Importantly, kidney cancer carries a relatively favourable prognosis compared with many other solid tumours [10], providing an extended window in which cachexia can influence outcomes and potentially be targeted therapeutically. Despite these observations, the contribution of bone metastases to cachexia in kidney cancer has not been systematically examined.

The purpose of this study was to determine (1) whether the presence of metastatic bone disease at diagnosis is associated with skeletal muscle loss over time and (2) whether metastatic bone disease or longitudinal skeletal muscle loss is associated with survival in patients with kidney cancer. We retrospectively analysed a large cohort of patients with localised disease, extraosseous metastases or osseous metastases at diagnosis, using artificial intelligence–assisted body composition analysis of CT scans to quantify longitudinal changes in muscle mass. We hypothesised that metastatic bone disease would be independently associated with accelerated muscle wasting and reduced survival.

## 2 Methods

### 2.1 Study Design and Patient Population

This was a retrospective cohort study approved by the Indiana University Institutional Review Board (IRB #13283). Adult patients (≥ 18 years) diagnosed with clear cell RCC between 2010 and 2019 were identified from an institutional cancer registry. Eligible patients were required to have a diagnostic computed tomography (CT) scan including the L1–L5 vertebral levels within 3 months of cancer diagnosis. Patients with fewer than 2 years of follow‐up were excluded unless death occurred earlier. A study flow diagram summarising inclusion and exclusion criteria is provided in Figure 1. To evaluate for potential selection bias across study subsets, baseline demographic, clinicopathologic, treatment and BMI characteristics were compared between the three primary analysis cohorts (initial BMI, longitudinal BMI and longitudinal CT body composition cohorts), with no statistically significant differences observed (Table S1).

**FIGURE 1:**
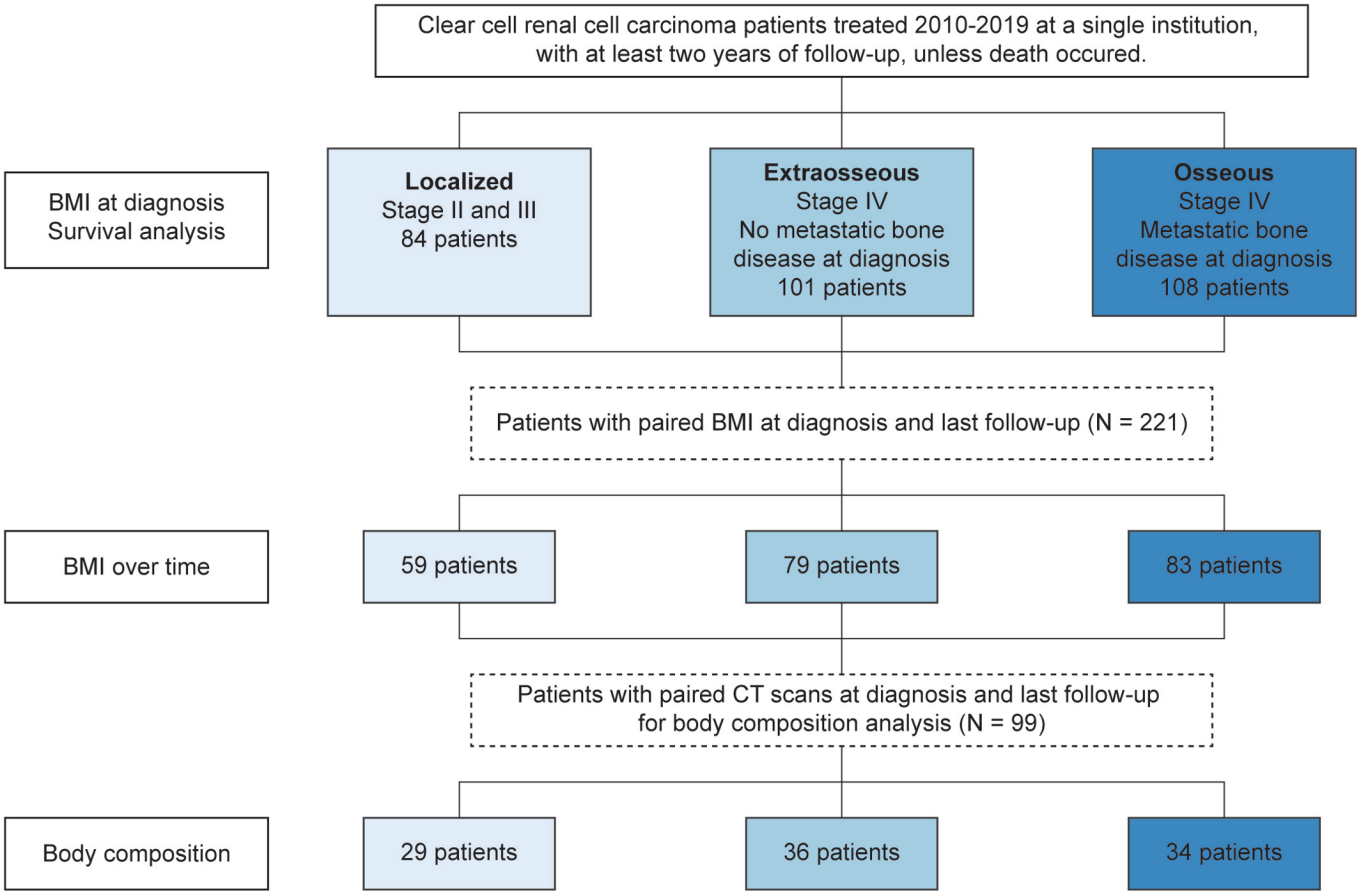
Flow diagram of patient selection and data availability across the study period. The diagram outlines the number of patients screened and included at various stages of the analysis.

### 2.2 Disease Classification and Clinical Variables

Patients were categorised into three disease groups at diagnosis: (i) localised disease, (ii) extraosseous metastatic disease without bone involvement and (iii) osseous metastatic disease with bone involvement. The number of metastatic sites was classified as none, 1–5, 6–10 or > 10. The presence of lung, liver, pancreatic or brain metastases at diagnosis and at last follow‐up was recorded. Additional clinical variables included age, sex, race, stage, systemic therapy (chemotherapy, immunotherapy) and use of bone‐modifying agents. Demographic and clinicopathologic characteristics are summarised in Table 1 and treatment characteristics and follow‐up in Table 2.

**TABLE 1:** Demographic characteristics, clinicopathologic characteristics and body mass index (BMI) at the time of diagnosis.

	Local (*N* = 84)	Extraosseous (*N* = 101)	Osseous (*N* = 108)	All cases (*N* = 293)	Overall *p*
Age (years)					
Median [min, max]	58.0 [25.0, 88.0]	62.0 [36.0, 88.0]	60.5 [26.0, 86.0]	60.0 [25.0, 88.0]	0.58
Sex					
Male	48 (57.1%)	69 (68.3%)	79 (73.1%)	196 (66.9%)	0.06
Female	36 (42.9%)	32 (31.7%)	29 (26.9%)	97 (33.1%)	
Race					
White	78 (92.9%)	91 (90.1%)	94 (87.0%)	263 (89.8%)	0.27
Black	4 (4.8%)	10 (9.9%)	11 (10.2%)	25 (8.5%)	
Other	2 (2.4%)	0 (0%)	3 (2.8%)	5 (1.7%)	
Stage					
2	38 (45.2%)	0 (0%)	0 (0%)	38 (13.0%)	—
3	46 (54.8%)	0 (0%)	0 (0%)	46 (15.7%)	
4	0 (0%)	101 (100%)	108 (100%)	209 (71.3%)	
Number of metastatic sites at diagnosis					
None	84 (100%)	0 (0%)	0 (0%)	84 (28.7%)	—
1–5	0 (0%)	56 (55.4%)	65 (60.2%)	121 (41.3%)	
6–10	0 (0%)	38 (37.6%)	15 (13.9%)	53 (18.1%)	
> 10	0 (0%)	7 (6.9%)	28 (25.9%)	35 (11.9%)	
Lung metastasis at diagnosis					
No	84 (100%)	22 (21.8%)	65 (60.2%)	171 (58.4%)	—
Yes	0 (0%)	79 (78.2%)	43 (39.8%)	122 (41.6%)	
Liver metastasis at diagnosis					
No	84 (100%)	73 (72.3%)	91 (84.3%)	248 (84.6%)	—
Yes	0 (0%)	28 (27.7%)	17 (15.7%)	45 (15.4%)	
Pancreas metastasis at diagnosis					
No	84 (100%)	99 (98.0%)	100 (92.6%)	283 (96.6%)	—
Yes	0 (0%)	2 (2.0%)	8 (7.4%)	10 (3.4%)	
Brain metastasis at diagnosis					
No	84 (100%)	86 (85.1%)	98 (90.7%)	268 (91.5%)	—
Yes	0 (0%)	15 (14.9%)	10 (9.3%)	25 (8.5%)	
Number of bone metastasis at diagnosis					
None	84 (100%)	101 (100%)	0 (0%)	185 (63.1%)	—
Solitary	0 (0%)	0 (0%)	62 (57.4%)	62 (21.2%)	
2–5	0 (0%)	0 (0%)	30 (27.8%)	30 (10.2%)	
6–10	0 (0%)	0 (0%)	7 (6.5%)	7 (2.4%)	
> 10	0 (0%)	0 (0%)	9 (8.3%)	9 (3.1%)	
BMI at diagnosis					
Median [min, max]	31.2 [17.2, 74.1]	28.4 [17.1, 64.8]	27.6 [16.9, 49.9]	28.4 [16.9, 74.1]	0.02^b^
Vital status at last contact					
Alive	68 (81.0%)	43 (42.6%)	38 (35.2%)	149 (50.9%)	< 0.01^a^, ^b^
Dead	16 (19.0%)	58 (57.4%)	70 (64.8%)	144 (49.1%)	
Diagnosis to last contact (days)					
Median [min, max]	1310 [14.0, 4260]	672 [12.0, 3680]	432 [3.00, 3090]	672 [3.00, 4260]	< 0.01^a^, ^b^, ^c^

*Note:* Superscript letters denote statistically significant pairwise comparisons following correction for multiple comparisons (adjusted *p* < 0.05). Statistical comparisons were not made for variables involving metastatic sites or treatment because these are inherently different between groups.

^a^ Local versus extraosseous.

^b^ Local versus osseous.

^c^ Extraosseous versus osseous.

**TABLE 2:** Demographic characteristics, clinicopathologic characteristics, treatment and body mass index (BMI) at last follow‐up.

	Local (*N* = 59)	Extraosseous (*N* = 79)	Osseous (*N* = 83)	All cases (*N* = 221)	Overall *p*
Number of metastatic sites at follow‐up					
None	45 (76.3%)	0 (0%)	0 (0%)	45 (20.4%)	—
1–5	11 (18.6%)	29 (36.7%)	29 (34.9%)	69 (31.2%)	
6–10	3 (5.1%)	29 (36.7%)	26 (31.3%)	58 (26.2%)	
> 10	0 (0%)	21 (26.6%)	28 (33.7%)	49 (22.2%)	
Lung metastasis at follow‐up					
No	51 (86.4%)	6 (7.6%)	40 (48.2%)	97 (43.9%)	—
Yes	8 (13.6%)	73 (92.4%)	43 (51.8%)	124 (56.1%)	
Liver metastasis at follow‐up					
No	58 (98.3%)	56 (70.9%)	54 (65.1%)	168 (76.0%)	—
Yes	1 (1.7%)	23 (29.1%)	29 (34.9%)	53 (24.0%)	
Pancreas metastasis at follow‐up					
No	59 (100%)	74 (93.7%)	70 (84.3%)	203 (91.9%)	—
Yes	0 (0%)	5 (6.3%)	13 (15.7%)	18 (8.1%)	
Brain metastasis at follow‐up					
No	57 (96.6%)	61 (77.2%)	70 (84.3%)	188 (85.1%)	—
Yes	2 (3.4%)	18 (22.8%)	13 (15.7%)	33 (14.9%)	
Number of bone metastasis at follow‐up					
None	56 (94.9%)	58 (73.4%)	1 (1.2%)	115 (52.0%)	—
Solitary	2 (3.4%)	10 (12.7%)	32 (38.6%)	44 (19.9%)	
2–5	1 (1.7%)	11 (13.9%)	34 (41.0%)	46 (20.8%)	
6–10	0 (0%)	0 (0%)	3 (3.6%)	3 (1.4%)	
> 10	0 (0%)	0 (0%)	13 (15.7%)	13 (5.9%)	
Chemotherapy					
No	47 (79.7%)	26 (32.9%)	40 (48.2%)	113 (51.1%)	—
Yes	12 (20.3%)	53 (67.1%)	43 (51.8%)	108 (48.9%)	
Immunotherapy					
No	51 (86.4%)	48 (60.8%)	58 (69.9%)	157 (71.0%)	—
Yes	8 (13.6%)	31 (39.2%)	25 (30.1%)	64 (29.0%)	
Bone‐modifying agents					
No	59 (100%)	76 (96.2%)	74 (89.2%)	209 (94.6%)	—
Yes	0 (0%)	3 (3.8%)	9 (10.8%)	12 (5.4%)	
BMI at follow‐up					
Median [min, max]	30.1 [16.0, 57.9]	27.7 [17.7, 53.4]	27.1 [16.7, 46.3]	28.0 [16.0, 57.9]	0.01^a^, ^b^
% change in BMI per year					
Median [min, max]	0.72 [−33.8, 28.2]	−2.59 [−66.1, 31.9]	−3.58 [−80.7, 61.8]	−0.91 [−80.7, 61.8]	<0.01^a^, ^b^
Interval between BMI (days)					
Median [min, max]	1320 [118, 4350]	887 [90.0, 3850]	476 [91.0, 2560]	880 [90.0, 4350]	<0.01^a^, ^b^, ^c^

*Note:* Superscript letters denote statistically significant pairwise comparisons following correction for multiple comparisons (adjusted *p* < 0.05). Statistical comparisons were not made for variables involving metastatic sites or treatment because these are inherently different between groups.

^a^ Local versus extraosseous.

^b^ Local versus osseous.

^c^ Extraosseous versus osseous.

### 2.3 Outcomes

The study outcomes included longitudinal changes in body composition and overall survival. Body composition was assessed using both chart‐derived body mass index (BMI) and CT‐based analysis of skeletal muscle, subcutaneous adipose tissue (SAT), visceral adipose tissue (VAT) and bone cross‐sectional area at the L3 vertebral level. Longitudinal change was expressed as annualised percent change for each compartment, and skeletal muscle loss was also categorised as no loss, 0%–20% loss or greater than 20% loss per year. Overall survival was defined as the time from diagnosis to death from any cause, with patients censored at the date of last known follow‐up.

### 2.4 Imaging Analysis

Body composition was quantified from diagnostic (within 3 months of diagnosis) and follow‐up (most recent) CT scans using Data Analysis Facilitation Suite (DAFS) (Voronoi Analytics, Vancouver, BC, Canada), an artificial intelligence–assisted segmentation platform. Analyses were performed at the midpoint of the L3 vertebra. Segmented compartments included skeletal muscle, SAT, VAT and bone. Visceral tissues, including tumour, were excluded. Automated outputs were manually reviewed by trained analysts for quality assurance. Representative images are shown in Figure 2.

**FIGURE 2:**
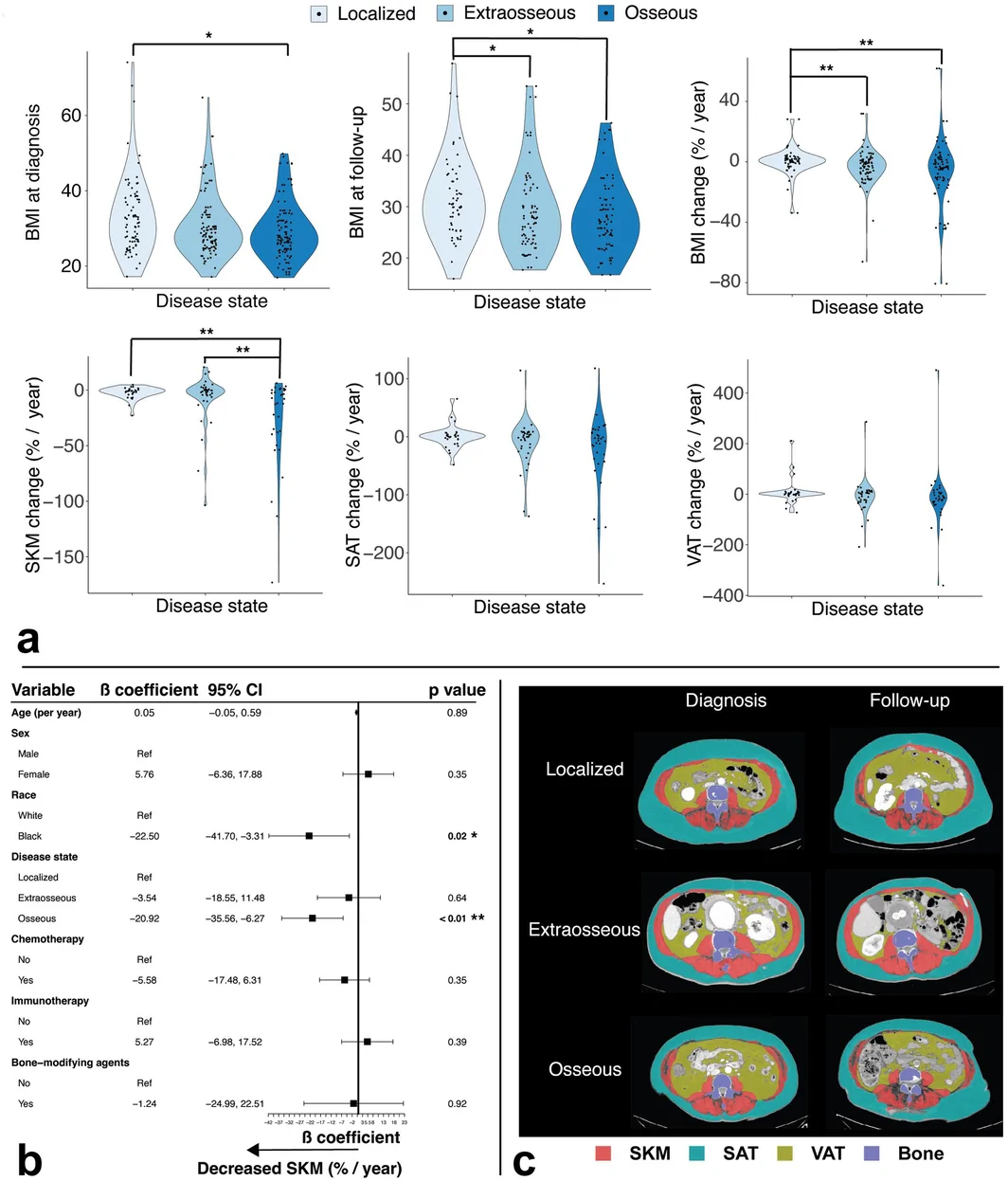
Osseous metastatic disease is associated with loss of skeletal muscle (SKM) in persons with renal cell carcinoma (RCC). Violin plots show changes in body composition overtime by disease state (a). Multiple linear regression (including covariates disease state, age at diagnosis, sex, race, chemotherapy, immunotherapy and use of bone‐modifying agents) shows osseous disease was associated with decreased SKM (b). *β* coefficients with 95% confidence intervals (CI) and *p* values for the multiple regression are included on the forest plot in b. Representative paired diagnostic and follow‐up CT images from the same patient within each disease group (selected to approximate the median SKM for that group) are shown in c, with red indicating SKM identified by artificial intelligence–assisted body composition analysis. Each datapoint represents an individual person in a. *p < 0.05. **p < 0.01. SAT, subcutaneous adipose tissue; SKM, skeletal muscle; VAT, visceral adipose tissue.

### 2.5 Statistical Analysis

Continuous variables were summarised as medians with ranges, as none passed normality testing. Group comparisons across disease states (localised, extraosseous metastases and osseous metastases) were performed using Kruskal–Wallis tests, with pairwise Wilcoxon rank‐sum tests adjusted for multiple comparisons using the Benjamini–Hochberg method. Categorical variables were compared using Fisher's exact tests, with pairwise Fisher's exact tests adjusted for multiple comparisons using the Benjamini–Hochberg method when appropriate.

The association of disease state with annualised skeletal muscle change (% per year) was examined using multiple variable linear regression models with covariates including age at diagnosis, sex, race, chemotherapy, immunotherapy and use of bone‐modifying agents. Model assumptions were assessed by residual diagnostics and variance inflation factors.

The association of disease state and body composition with overall survival was examined using Kaplan–Meier methods and compared with log‐rank tests. Cox proportional hazards models were also constructed to evaluate the association of disease state and annualised changes in skeletal muscle with survival, adjusted for age, chemotherapy, immunotherapy and annualised changes in SAT and VAT. Proportional hazards assumptions were tested using Schoenfeld residuals. Overall survival was defined from the date of diagnosis to death from any cause, with survivors censored at last follow‐up.

Data visualisation included violin plots with overlaid jittered data points to display distributions of BMI and body composition measures across disease states, as well as Kaplan–Meier survival curves with risk tables. Figures were generated in R and finalised for publication using Adobe Illustrator (version 28.0.0; Adobe Inc., San Jose, CA).

All analyses were conducted in R (version 4.4.1; R Foundation for Statistical Computing, Vienna, Austria) using the packages *tidyverse*, *table1*, *survival*, *survminer*, *ggplot2*, *ggfortify*, *car*, *forestplot*, *mice* and *broom*.

## 3 Results

### 3.1 Body Composition Changes by Disease State

All 293 patients had BMI values available at diagnosis (84 localised, 101 extraosseous metastases, 108 osseous metastases). Median BMI at diagnosis was 31.2 for localised disease, 28.4 for extraosseous metastases and 27.6 for osseous metastases (*p* < 0.05 for comparison between localised and osseous groups; Figure 2a; Table 1). BMI overtime was available for 221 patients, for which BMI increased slightly in localised disease (+0.7% per year), declined in extraosseous disease (−2.6% per year) and declined most rapidly in osseous disease (−3.6% per year; *p* < 0.01 for comparison between localised and osseous groups; Figure 2a; Table 2).

Ninety‐nine patients (29 localised, 36 extraosseous, 34 osseous) had serial CT scans available for analysis of body composition. At baseline, median skeletal muscle cross‐sectional area at L3 was similar at 165 cm^2^ for localised disease, 155 cm^2^ for extraosseous metastases and 151 cm^2^ for osseous metastases (*p* = 0.48 overall). Median annualised skeletal muscle change was −0.8% in localised disease and −1.2% in extraosseous disease, compared with −7.5% in osseous disease (*p* < 0.01 for both comparisons; Figure 2a,c; Table 3).

**TABLE 3:** Computed tomography (CT) body composition analysis at diagnosis, at follow‐up and over time.

	Local (*N* = 29)	Extraosseous (*N* = 36)	Osseous (*N* = 34)	All cases (*N* = 99)	Overall *p*
Muscle at diagnosis					
Median [min, max]	165 [89.5, 257]	155 [82.3, 257]	151 [90.2, 275]	155 [82.3, 275]	0.48
Muscle at follow‐up					
Median [min, max]	166 [92.4, 202]	144 [80.7, 234]	121 [72.4, 242]	140 [72.4, 242]	0.07
Muscle change (%/year)					
Median [min, max]	−0.78 [−22.9, 4.84]	−1.23 [−104, 20.7]	−7.52 [−173, 6.23]	−1.96 [−173, 20.7]	0.01^b^, ^c^
Bone at diagnosis					
Median [min, max]	12.3 [5.25, 36.7]	16.2 [6.83, 36.7]	17.4 [5.76, 47.8]	15.5 [5.25, 47.8]	0.28
Bone at follow‐up					
Median [min, max]	15.3 [8.29, 32.9]	17.4 [8.92, 29.1]	15.2 [6.49, 36.7]	16.2 [6.49, 36.7]	0.77
Bone change (%/year)					
Median [min, max]	2.00 [−49.6, 116]	0.535 [−175, 402]	−3.59 [−179, 1140]	0.468 [−179, 1140]	0.08
SAT at diagnosis					
Median [min, max]	236 [84.7, 803]	220 [68.2, 789]	229 [39.4, 685]	229 [39.4, 803]	0.78
SAT at follow‐up					
Median [min, max]	253 [41.9, 957]	211 [0.673, 641]	210 [29.6, 616]	238 [0.673, 957]	0.34
SAT change (%/year)					
Median [min, max]	0.576 [−48.1, 65.4]	−0.326 [−137, 114]	−5.07 [−253, 118]	−0.508 [−253, 118]	0.52
VAT at diagnosis					
Median [min, max]	185 [10.1, 562]	153 [10.4, 418]	160 [7.23, 566]	167 [7.23, 566]	0.76
VAT at follow‐up					
Median [min, max]	200 [27.4, 507]	127 [1.68, 517]	170 [6.10, 436]	156 [1.68, 517]	0.53
VAT change (%/year)					
Median [min, max]	1.47 [−72.4, 211]	1.03 [−208, 286]	−15.6 [−361, 491]	−1.10 [−361, 491]	0.21
Interval between CT scans (days)					
Median [min, max]	1140 [206, 4200]	858 [56.0, 3850]	464 [47.0, 2070]	862 [47.0, 4200]	<0.01^a^, ^b^, ^c^

*Note:* All values represent the area of skeletal muscle, bone, subcutaneous adipose tissue (SAT) and visceral adipose tissue (VAT) in square centimetre at mid‐L3 level. Superscript letters denote statistically significant pairwise comparisons following correction for multiple comparisons (adjusted *p* < 0.05).

^a^ Local versus extraosseous.

^b^ Local versus. osseous.

^c^ Extraosseous versus osseous.

SAT declined by −5.1% per year in osseous disease, compared with −0.3% in extraosseous and +0.6% in localised disease (*p* = 0.53 overall). VAT declined by −15.6% per year in osseous disease compared with +1.0% in extraosseous and +1.5% in localised disease (*p* = 0.21 overall). Bone cross‐sectional area decreased by −3.6% per year in osseous disease but increased by +2.0% in localised and +0.5% in extraosseous disease (*p* = 0.08 overall) (Figure 2a,c; Table 3).

In multiple variable linear regression analysis, adjusting for age, sex, race, chemotherapy, immunotherapy and use of bone‐modifying agents, osseous metastases at diagnosis were independently associated with an additional −20.9% loss of skeletal muscle per year compared with localised disease (*β* coefficient of −20.9; 95% confidence interval [CI]: −35.6 to −6.3; *p* < 0.01) (Figure 2b).

### 3.2 Survival by Disease State and Muscle Loss

Median follow‐up for the cohort was 672 days (range 3–4260 days). Kaplan–Meier analysis demonstrated worse survival for patients with osseous metastases compared with localised or extraosseous disease at diagnosis (total events [deaths] = 144; *p* < 0.01 compared with localised disease; Figure 3a). One‐year overall survival was 53.3% for patients with osseous disease (95% CI: 44.7 to 63.7%), 88.1% for localised disease (95% CI 81.4 to 95.3%) and 72.3% for extraosseous disease (95% CI: 64.0 to 81.5%). Five‐year overall survival was 30.7% for patients with osseous disease (95% CI: 21.9 to 43.1%), 80.0% for localised disease (95% CI: 71.0 to 89.8%) and 27.9% for extraosseous disease (95% CI: 18.0 to 43.3%).

**FIGURE 3:**
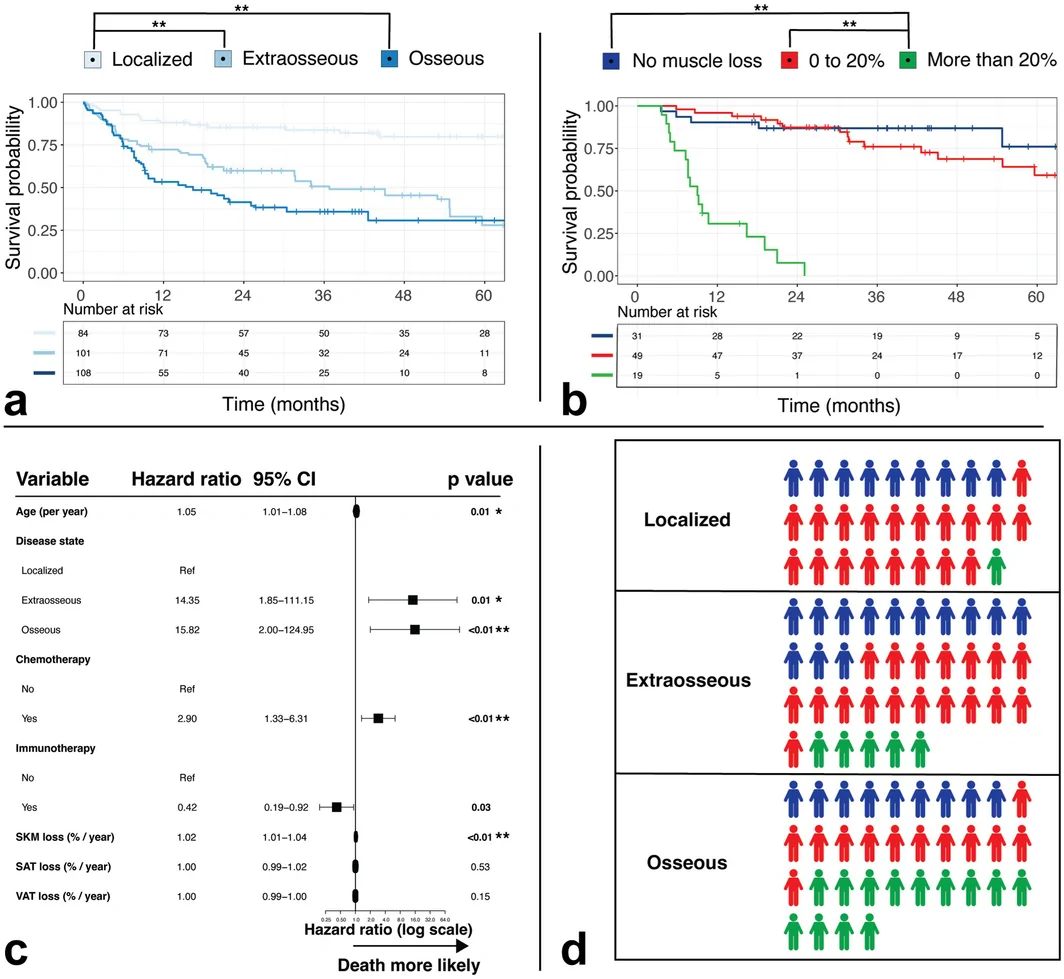
Osseous metastatic disease and loss of skeletal muscle (SKM) are associated with survival in persons with renal cell carcinoma (RCC). Data summarise findings from a retrospective chart review at my institution. Data obtained from medical records (clinical) and artificial intelligence–assisted analysis of CT scans (body composition). Kaplan–Meier curves show that patients with osseous metastatic disease have worse survival (a) than other disease states and that those with more than 20% loss of SKM per year have worse survival than those with no or 0%–20% SKM loss per year (b). Multiple Cox regression analysis (including covariates disease state, age at diagnosis, chemotherapy, immunotherapy, SKM, SAT and VAT loss) demonstrated that annualised skeletal muscle loss and osseous disease were associated with increased risk of death (c). Hazard ratios with 95% confidence intervals (CI) and *p* values for the multiple regression are included on the forest plot in c. Visual representation of individual persons in each disease category (localised, extraosseous metastasis and osseous metastasis) with no muscle loss (blue), 0%–20% (red), and more than 20% (green) (d). *p < 0.05. **p < 0.01. SAT, subcutaneous adipose tissue; SKM, skeletal muscle; VAT, visceral adipose tissue.

Kaplan–Meier analysis stratified by skeletal muscle loss also showed significant differences in survival (*p* < 0.01 for patients with > 20% annualised muscle loss compared with patients with 0%–20% loss or no loss; Figure 3b). One‐year overall survival was 30.7% for patients with > 20% muscle loss per year (95% CI: 15.4% to 61.1%), 90.3% for no muscle loss (95% CI 80.5% to 100%) and 95.9% for 0%–20% loss (95% CI: 90.5% to 100%). Five‐year overall survival was 0% for patients with > 20% muscle loss per year (95% CI: not applicable), 76.0% for no muscle loss (95% CI: 56.5% to 100%) and 59.2% for 0%–20% loss (95% CI: 43.7% to 80.3%).

In multiple variable Cox regression analysis, adjusting for age, chemotherapy, immunotherapy, subcutaneous and VAT loss, both disease state and skeletal muscle loss remained significantly associated with overall survival. The presence of osseous metastases at diagnosis was associated with a higher hazard of death compared with localised disease (hazard ratio [HR] of 15.8; 95% CI: 2.00 to 124.95; *p* < 0.01). Skeletal muscle loss was also associated with increased hazard of death (HR of 1.02 for each % loss per year; 95% CI: 1.01 to 1.04; *p* < 0.01) (Figure 3c). A visual representation of individual persons in each disease group (localised, extraosseous and osseous) and their corresponding degree of muscle loss is depicted in Figure 3d with 38.2% (13/34) of patients in the osseous group having > 20% muscle loss per year, compared with 3.4% (1/29) in the localised group and 13.9% (5/36) in the extraosseous group.

## 4 Discussion

In this retrospective cohort of patients with kidney cancer, osseous metastatic disease was associated with greater longitudinal loss of skeletal muscle and inferior survival, compared with patients with localised or extraosseous metastatic disease. Severe skeletal muscle loss (> 20% per year) identified a subgroup with markedly worse outcomes, consistent with the growing recognition of cachexia as a determinant of prognosis in cancer. These results extend prior observations in kidney cancer that link baseline muscle status and other body‐composition features to outcomes, and they align with contemporary work emphasising the role of the tumour microenvironment and multi‐organ interactions, including bone, in the development of cancer cachexia.

Body‐composition analyses demonstrated significantly greater skeletal muscle loss in patients with bone metastases compared with those with localised or extraosseous disease. In multiple variable linear regression, the presence of osseous metastases was independently associated with greater annualised skeletal muscle decline after adjustment for demographic and treatment covariates. To our knowledge, this is the first study to demonstrate an association between bone metastases and muscle loss for kidney cancer. No statistically significant differences were observed for subcutaneous or VAT, although a trend toward reduced bone cross‐sectional area was noted in the osseous metastatic group. This observation aligns with prior reports describing bone loss in the setting of cancer and suggests a potential interaction between skeletal metastases and systemic musculoskeletal changes [11]. Although this study was not designed to establish causation or mechanism, the results provide clinical context supporting further investigation of tumour–bone–muscle interactions. Preclinical data, including work by Waning and Guise showing that TGF‐β released from bone metastases disrupts ryanodine receptor function and impairs muscle contractility in murine models, highlight plausible biologic pathways that warrant translational study in patients [7].

Survival patterns by disease state in this cohort were consistent with prior reports: Patients with osseous metastases had worse overall survival than those with localised or extraosseous disease [12, 13]. Severe skeletal muscle loss also emerged as a strong factor associated with mortality. This largely aligns with recent literature showing that body‐composition features—particularly low or declining skeletal muscle—track with inferior survival outcomes in localised [9, 14, 15] and metastatic [9, 16, 17, 18] kidney cancer, and mirrors emerging prospective evidence in other cancers [19]. Conversely, one study examining muscle loss in metastatic RCC reported that low muscle mass at diagnosis was associated with better survival [20]. Another showed that loss of muscle 3–4 months after treatment initiation was associated with improved survival [21]. All of these prior studies are limited because body composition was analysed only at diagnosis or over short 3–4‐month interval after starting systemic therapy. Longitudinal assessment of body composition over clinically relevant intervals, such as the two‐year window in this study, reduces the influence of short‐term fluctuations around treatment initiation and provides a more accurate reflection of tumour‐ and host‐specific drivers of cachexia progression. Notably, analyses from the TRACERx (non‐small‐cell lung cancer) program have shown that longitudinal loss of skeletal muscle and/or adipose tissue is associated with worse survival, underscoring the generalisability of dynamic body‐composition change as a prognostic signal across malignancies [22]. In response, efforts are emerging to mitigate muscle loss through structured physical activity and rehabilitation programs, alongside ongoing research to identify therapeutic targets aimed at reversing cancer‐associated cachexia and improving survival [19, 23]. To date, such strategies have not been systematically investigated in kidney cancer, highlighting an important opportunity for future study.

This study has limitations. It is a single‐institution retrospective cohort and subject to selection and information bias inherent to chart review. Although the overall sample size was moderate, only a subset had serial CT scans suitable for longitudinal body‐composition analysis, which may limit power for some subgroup and regression analyses. In addition, other CT‐based metrics, including skeletal muscle density, skeletal muscle index and volumetric analyses were not available and may provide complementary information in future studies. Additionally, follow‐up intervals differed substantially between disease groups, with shorter follow‐up in patients with metastatic disease, particularly those with osseous metastases, largely due to reduced survival. These differences in follow‐up duration may have influenced longitudinal body‐composition analyses. To partially address this limitation, changes in BMI and CT‐derived body‐composition metrics were annualised. However, patients with metastatic disease and osseous metastases likely represent more advanced systemic disease states overall, and the extent to which bone metastases directly contribute to skeletal muscle loss, versus simply associating with more late‐stage disease, remains uncertain. Prospective longitudinal studies with standardised imaging intervals and mechanistic investigations will be needed to better define causal tumour–bone–muscle relationships. Residual confounding from unmeasured variables (e.g., performance status, comorbidity burden, precise timing/intensity and type of systemic therapies, nutritional or exercise interventions) is possible. Nonetheless, the magnitude and consistency of body‐composition differences by disease state, and the independent association of severe muscle loss with mortality, motivate validation in broader, multi‐institutional cohorts.

## 5 Conclusions

Metastatic bone disease in kidney cancer is associated with accelerated skeletal muscle loss, and severe muscle wasting identifies patients at high risk for death. These findings reinforce cachexia as a clinically meaningful and potentially modifiable factor influencing survival. Future work should (i) replicate these results in larger, prospective cohorts; (ii) integrate serial body‐composition measures with functional and inflammatory biomarkers; (iii) develop preclinical models to interrogate tumour–bone–muscle pathways; and (iv) test targeted cachexia strategies in populations most likely to benefit. Given the frequency of bone metastases in kidney cancer and the extended treatment window, metastatic kidney cancer with skeletal involvement may be a rational setting for early trials of cachexia‐directed interventions.

## Ethics Statement

Ethical approval for this research was granted by the Indiana University School of Medicine Institutional Review Board (IRB13283) and was conducted according to the Declaration of Helsinki. The requirement for signed informed consent was waived. The authors of this manuscript certify that they comply with the ethical guidelines for authorship and publishing in the *Journal of Cachexia, Sarcopenia, and Muscle* [24].

## Conflicts of Interest

The authors declare no conflicts of interest.

## Supporting information


**TABLE S1:** Demographic and clinicopathologic characteristics at the time of diagnosis and treatment by analysis cohort.
