# Supplementary material for: Metastatic Bone Disease Is Associated With Longitudinal Muscle Loss and Reduced Survival in Kidney Cancer
Source: J Cachexia Sarcopenia Muscle. 2026 Oct 1;17(5):e70400. doi: 10.1002/jcsm.70400 (PMC13631380; doi:10.1002/jcsm.70400)
Supplement: Supplementary file 1 [file JCSM-17-e70400-s001.docx]

**Supplemental Table 1.** Demographic and clinicopathologic at time of diagnosis and treatment by analysis cohort.

|  | **Initial BMI (N=293)** | **BMI over time (N=221)** | **CT over time (N=99)** | **Overall p-value** |
| --- | --- | --- | --- | --- |
| **Age (years)** |  |  |  |  |
| Median [Min, Max] | 60.0 [25.0, 88.0] | 59.0 [25.0, 86.0] | 59.0 [37.0, 84.0] | 0.65 |
| **Sex** |  |  |  |  |
| Male | 196 (66.9%) | 152 (68.8%) | 67 (67.7%) | 0.91 |
| Female | 97 (33.1%) | 69 (31.2%) | 32 (32.3%) |  |
| **Race** |  |  |  |  |
| White | 263 (89.8%) | 199 (90.0%) | 89 (89.9%) | 0.50 |
| Black | 25 (8.5%) | 21 (9.5%) | 9 (9.1%) |  |
| Other | 5 (1.7%) | 1 (0.5%) | 1 (1.0%) |  |
| **Stage** |  |  |  |  |
| 2 | 38 (13.0%) | 28 (12.7%) | 11 (11.1%) | 0.90 |
| 3 | 46 (15.7%) | 31 (14.0%) | 18 (18.2%) |  |
| 4 | 209 (71.3%) | 162 (73.3%) | 70 (70.7%) |  |
| **Number of metastatic sites at diagnosis** |  |  |  |  |
| None | 84 (28.7%) | 59 (26.7%) | 29 (29.3%) | 0.90 |
| 1-5 | 121 (41.3%) | 103 (46.6%) | 43 (43.4%) |  |
| 6-10 | 53 (18.1%) | 39 (17.6%) | 18 (18.2%) |  |
| > 10 | 35 (11.9%) | 20 (9.0%) | 9 (9.1%) |  |
| **Lung metastasis at diagnosis** |  |  |  |  |
| No | 171 (58.4%) | 131 (59.3%) | 60 (60.6%) | 0.92 |
| Yes | 122 (41.6%) | 90 (40.7%) | 39 (39.4%) |  |
| **Liver metastasis at diagnosis** |  |  |  |  |
| No | 248 (84.6%) | 191 (86.4%) | 85 (85.9%) | 0.86 |
| Yes | 45 (15.4%) | 30 (13.6%) | 14 (14.1%) |  |
| **Pancreas metastasis at diagnosis** |  |  |  |  |
| No | 283 (96.6%) | 212 (95.9%) | 96 (97.0%) | 0.93 |
| Yes | 10 (3.4%) | 9 (4.1%) | 3 (3.0%) |  |
| **Brain metastasis at diagnosis** |  |  |  |  |
| No | 268 (91.5%) | 202 (91.4%) | 92 (92.9%) | 0.93 |
| Yes | 25 (8.5%) | 19 (8.6%) | 7 (7.1%) |  |
| **Number of bone metastasis at diagnosis** |  |  |  |  |
| None | 185 (63.1%) | 138 (62.4%) | 65 (65.7%) | 0.99 |
| Solitary | 62 (21.2%) | 53 (24.0%) | 22 (22.2%) |  |
| 2-5 | 30 (10.2%) | 20 (9.0%) | 8 (8.1%) |  |
| 6-10 | 7 (2.4%) | 4 (1.8%) | 1 (1.0%) |  |
| > 10 | 9 (3.1%) | 6 (2.7%) | 3 (3.0%) |  |
| **Chemotherapy** |  |  |  |  |
| No | 171 (58.4%) | 113 (51.1%) | 53 (53.5%) | 0.25 |
| Yes | 122 (41.6%) | 108 (48.9%) | 46 (46.5%) |  |
| **Immunotherapy** |  |  |  |  |
| No | 227 (77.5%) | 157 (71.0%) | 66 (66.7%) | 0.06 |
| Yes | 66 (22.5%) | 64 (29.0%) | 33 (33.3%) |  |
| **Bone-modifying agents** |  |  |  |  |
| No | 281 (95.9%) | 209 (94.6%) | 93 (93.9%) | 0.60 |
| Yes | 12 (4.1%) | 12 (5.4%) | 6 (6.1%) |  |
| **BMI at diagnosis** |  |  |  |  |
| Median [Min, Max] | 28.4 [16.9, 74.1] | 27.9 [17.2, 74.1] | 28.2 [17.8, 54.4] | 0.78 |
